# Granulomatosis With Polyangiitis: A Case of a 62-Year-Old Female Presenting With a Fever of Unknown Origin

**DOI:** 10.7759/cureus.107340

**Published:** 2026-04-19

**Authors:** Muhammad Abrar Ul Haq, Bilal Saeed, Yasir Ali, Ayesha Hina, Paul Eoin Cotter

**Affiliations:** 1 Geriatric Medicine, St. Luke’s General Hospital, Kilkenny, IRL; 2 Internal Medicine, St. Luke’s General Hospital, Kilkenny, IRL; 3 Emergency Medicine, St. Luke’s General Hospital, Kilkenny, IRL

**Keywords:** c-anca/proteinase 3-positive granulomatosis with polyangiitis, differential for fever of unknown origin, granulomatosis with polyangiitis (gpa), rheumatological diseases, small vessel vasculitis

## Abstract

Fever of unknown origin (FUO) continues to present diagnostic challenges despite advancements in medical science. Granulomatosis with polyangiitis (GPA), a rare antineutrophil cytoplasmic antibody (ANCA)-associated small-vessel vasculitis characterized by necrotizing granulomas, is an uncommon but important cause of FUO. This case report describes a 62-year-old female presenting with prolonged fever, malaise, and nonspecific systemic symptoms, ultimately diagnosed with GPA after extensive investigations, including positive C-ANCA and anti-proteinase-3 serology and imaging revealing a cavitating lung lesion. The patient’s symptoms improved following immunosuppressive therapy with corticosteroids and rituximab. This case highlights the necessity of considering vasculitis, particularly GPA, early in the differential diagnosis of FUO to enable timely treatment and prevent serious complications.

## Introduction

In the present world of enormous advances in the medical and technological fields, fever of unknown origin (FUO) still presents challenges to internists [1]. FUO was defined in 1961 as a febrile disease lasting more than three weeks with fever documented as 101°F at multiple times despite one week of hospital investigations [2]. In 2003, Knockaert et al. suggested a qualitative definition of FUO as a disease in which, after a certain period of standard inpatient and outpatient investigations, no reasonable diagnosis has been identified [3]. Five chief etiologies may account for FUO, namely, infectious, inflammatory, miscellaneous, malignant, and undiagnosed [4]. Non-infectious inflammatory conditions may comprise 20.9% of FUO cases [5]. A study from Serbia showed that rheumatological diseases caused 25.6% of the FUO cases [6].

Granulomatosis with polyangiitis (GPA) is a vasculitis disease affecting small and medium vessels, accompanied by necrotizing granuloma [7]. The presence of antineutrophilic cytoplasmic antibodies (ANCAs) directed against proteinase-3 (C-ANCA anti-PR3) is the key diagnostic marker of GPA [8]. GPA mainly targets the upper and lower respiratory tracts and kidneys, but other systems and organs may be involved [9]. Its incidence is equal among males and females, and it most commonly presents in middle age [10]. GPA has the potential to present as FUO and can have major complications if it remains undiagnosed. This case report highlights the importance of vasculitis, specifically GPA, as an early differential of FUO to detect and treat the pathology early.

## Case presentation

A 62-year-old female with no past medical history and no regular medications presented to the Acute Medical Assessment Unit with chief complaints of malaise, generalized body pains, lethargy, nighttime drenching sweats, and intermittent fever for the past four weeks. She visited a general practitioner and was prescribed a course of amoxicillin and paracetamol; however, her condition did not improve. Of note, she had a single kidney, as six years earlier, she had donated one of her kidneys to her husband for transplant. She denied any history of systemic symptoms of headache, confusion, photophobia, rashes, small joint pains, abdominal pain, chest pain, hemoptysis, diarrhea, burning micturition, or hematuria. She reported a loss of appetite. She had no known allergies.

**Figure 1:**
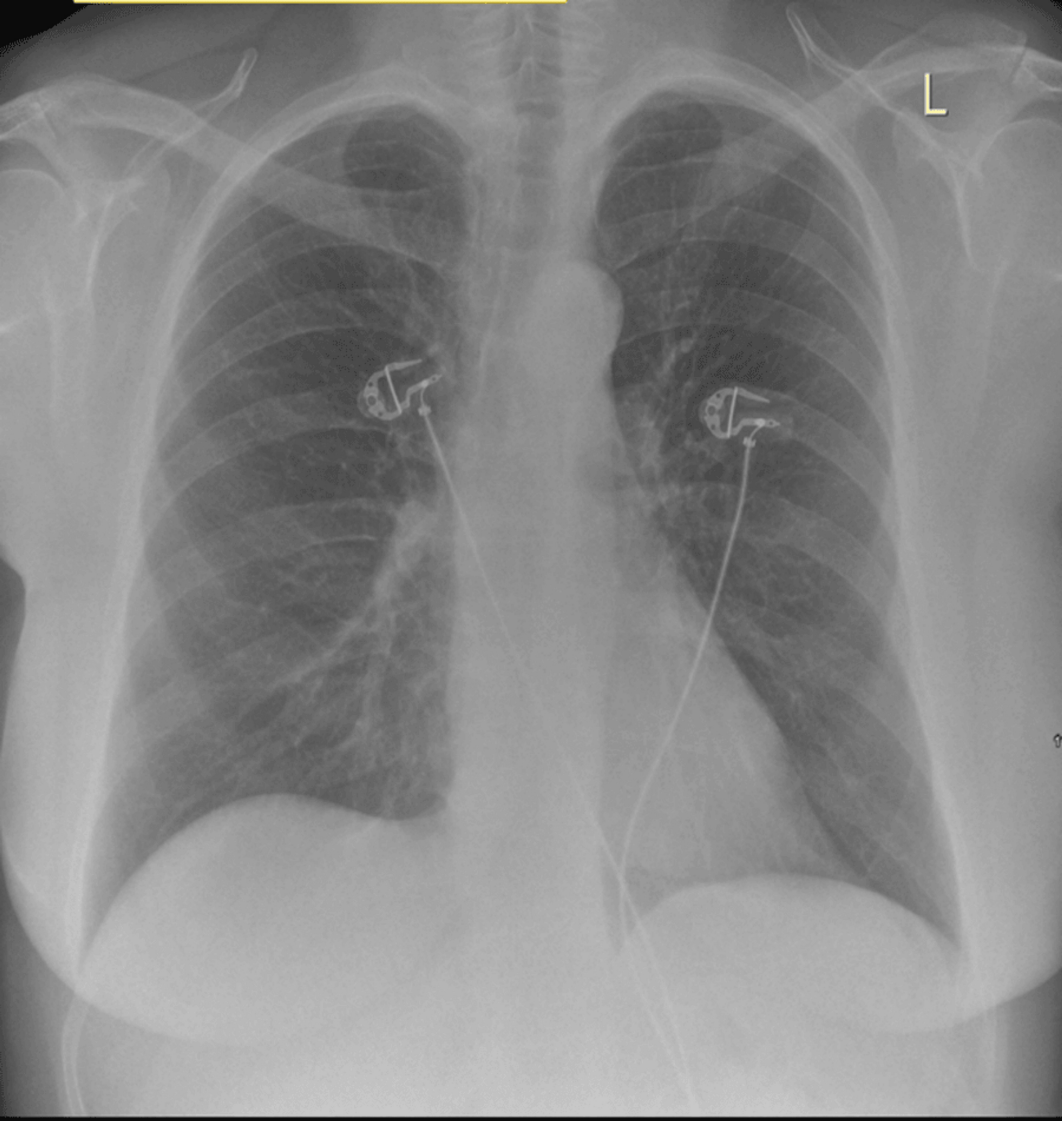
Chest X-ray on presentation showing no signs of consolidation or inflammatory infiltrates.

On general physical examination, she had a temperature of 36.8°C, a heart rate of 84 beats/minute, a blood pressure of 148/65 mmHg, and oxygen saturation of 96% on room air. There was no evidence of pallor, rashes, jaundice, nail changes, cyanosis, or lymphadenopathy. Her chest examination revealed normal vesicular breathing bilaterally. Her cardiovascular examination did not show any murmurs, raised jugular venous pressure, or peripheral edema. Her abdominal examination revealed a non-tender abdomen and no signs of hepatosplenomegaly or any other visceromegaly. Routine investigations were performed, as illustrated in Table 1 and Figure 1.

**Table 1:** Initial investigations.

Laboratory test	Results	Reference range
pH	7.43	7.32–7.42
PCO₂	4.8 kPa	4.6–6.4 kPa
Lactate	0.9 mmol/L	0.9–1.7 mmol/L
Bicarbonate	24 mmol/L	22–29 mmol/L
Urea	5.2 mmol/L	2.5–7.8 mmol/L
Creatinine	96 mmol/L	44–80 mmol/L
Alanine aminotransferase	21 IU/L	5–33 IU/L
Bilirubin	7.7 Umol/L	2.0–21 Umol/L
Gamma-glutamyl transferase	36 IU/L	3–40 IU/L
Alkaline phosphatase	86 IU/L	30–130 IU/L
Calcium	2.52 mmol/L	2.20–2.60 mmol/L
C-reactive protein	111 mg/L	0–5 mg/L
Creatine phosphokinase	117 IU/L	25–200 IU/L
White blood cell count	8.9 × 10⁹/L	4–10 × 10⁹/L
Hemoglobin	11.4 g/dL	12–15.5 g/dL
Platelets	345 × 10⁹/L	150–450 × 10⁹/L
Eosinophil	0.09 × 10⁹/L	0.02–0.5 × 10⁹/L
Glucose	7.8 mmol/L	3.0–6.0 mmol/L
Brain natriuretic peptide	32 pg/mL	0–100 pg/mL
Troponin	<0.01 µg/mL	0–0.05 µg/mL
COVID-19	Negative	Negative
Flu swab	Negative	Negative
Respiratory syncytial virus swab	Negative	Negative
Urine microscopy, culture, and sensitivity	White blood cell = 0, red blood cell = 0, epithelial cells = 0, no growth	White blood cells and red blood cells less than and no significant growth
Urine dip	Nitrite (-) leukocyte (-) protein (-)	Negative
Ferritin	510 µg/L	13-150 µg/L
Folate	7.3 ng/mL	3.0–26.8 ng/mL
Vitamin B12	287 pg	197–771 pg
Erythrocyte sedimentation rate	118 mm/hour	1–20 mm/hour
Lactate dehydrogenase	140 IU/L	10–250 IU/L
Stool occult blood	Not detected	Not detected

Impression

The patient was admitted with an initial impression of inflammation from a likely infective source. Oral antibiotics were started, and a workup was started for other sources of inflammation, and an abdominal ultrasound was ordered to rule out any abdominal source of infection.

Further progress

Over the next two days, the patient remained vitally stable but had ongoing malaise. Daily repeated blood tests showed persistently raised C-reactive protein (CRP). On the fourth day of admission, despite IV antibiotics, she spiked a temperature and was reviewed over the weekend with repeat blood cultures. On the sixth day of admission, the patient’s malaise continued, and she described left ear pain and a headache. Bedside otoscopy did not show any otorrhea, and the tympanic membrane and hearing test were normal. A CT of the brain and sinuses was reported as normal. Ultrasound of the abdomen was also reported to be normal. A further thorough history revealed that the patient had been on a recent cruise ship traveling to Portugal in the past two months. Furthermore, she lived on farms and had exposure to animals. Her father had died of tuberculosis-related complications while she was still young.

Differential diagnosis

**Figure 2:**
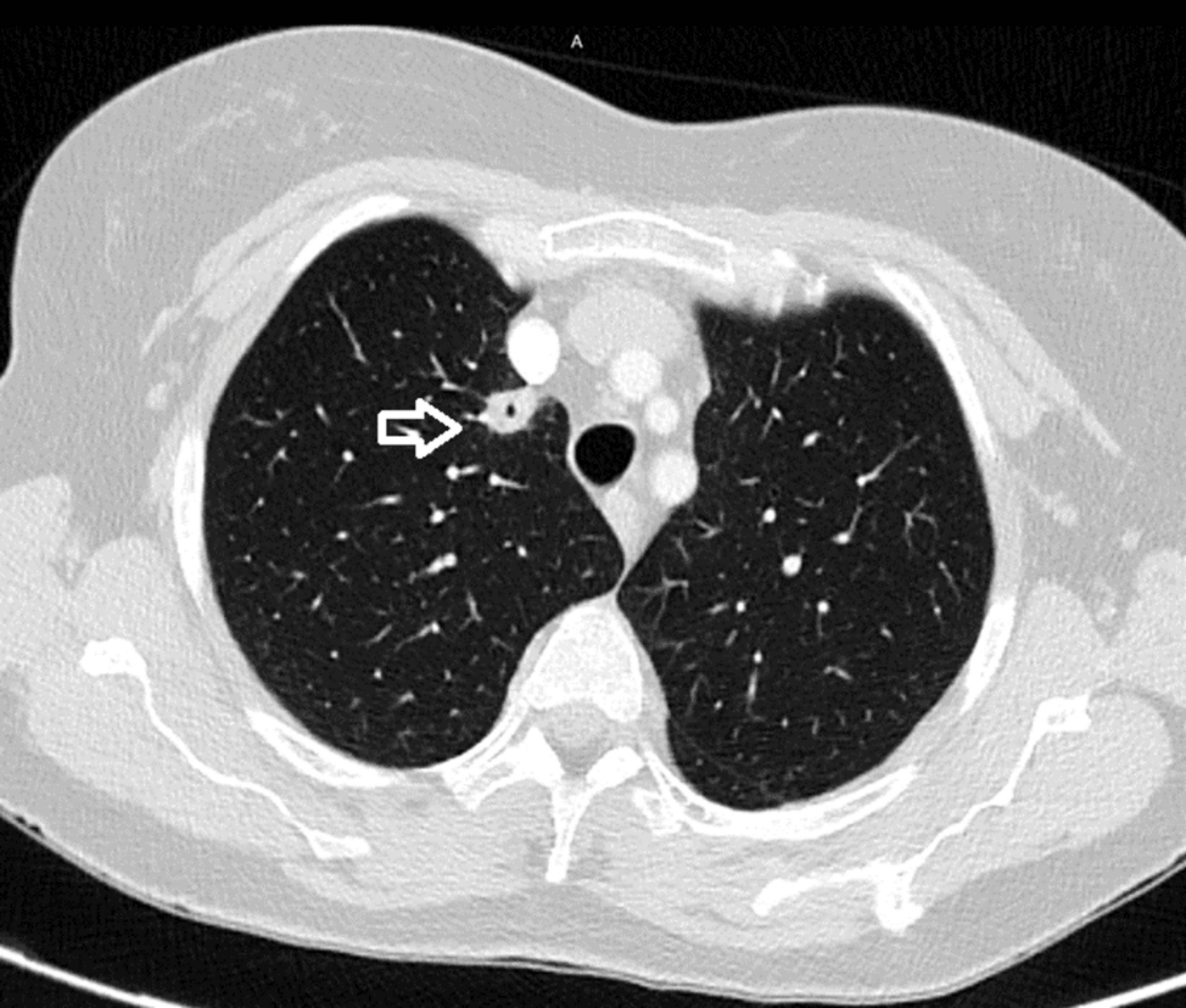
Axial CT imaging of the lung showing a cavitating lesion in the right upper lobe (white arrow).

**Figure 3:**
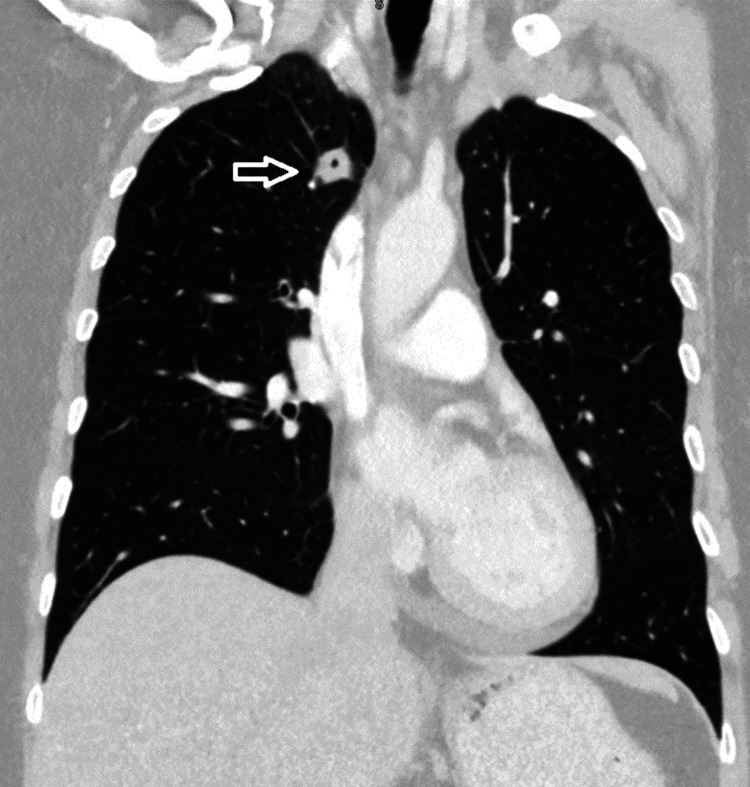
Coronal CT imaging of the lung showing a cavitating lesion in the right upper lobe (white arrow).

Our differential diagnoses at this point were atypical infections such as tuberculosis, Q fever, brucellosis, leptospirosis, or non-infectious inflammatory disorders or malignancy, such as lymphoma. For further workup, a CT of the thorax, abdomen, and pelvis (CT TAP) with contrast was ordered for any source of inflammation or occult malignancy. Quantiferon, *Coxiella burnetii* serology, leptospira serology, and *Brucella* serology were sent. Furthermore, HIV, hepatitis B, C, and cytomegalovirus serology were also ordered along with connective disease screening. On the seventh day of admission, the patient’s CT TAP was suggestive of a cavitating lesion in the right upper lobe (Figures 2, 3). Given the high suspicion of tuberculosis contact, the patient was isolated, and a respiratory opinion was sought for bronchoscopy, and sputum cultures for acid-fast bacilli were sent.

Diagnosis

On day nine of admission, the patient’s autoimmune serology was suggestive of C-ANCA-positive and anti-PR3 levels greater than 100 IU/mL (Table 2). At this point, the patient’s QuantiFERON results were reported as indeterminate. An inpatient bronchoscopy revealed normal airways. A biopsy of the vastus lateralis muscle was performed for a vasculitis workup, which was reported as normal. Urine albumin-creatinine ratio was performed for renal involvement, which was normal. A provisional diagnosis of GPA was made. The patient’s case was discussed with the rheumatology department, and the patient was started on a pulse dose of IV steroids for three days with a tapering oral steroid course and follow-up in the rheumatology clinic.

**Table 2:** Investigations results.

Investigation	Results
Cytomegalovirus IgM	Not detected
Cytomegalovirus IgG	Not detected
Epstein–Barr virus viral-capsid antigen IgM	Not detected
Epstein–Barr virus viral-capsid antigen IgG	Detected
Epstein–Barr virus nuclear antigen 1 IgG	Detected
Hepatitis B surface antigen Qual II	Not detected
Hepatitis A virus IgM	Not detected
Anti-hepatitis C virus	Not detected
HIV Ag/Ab combo	Not detected
Rheumatoid factor	18 IU/mL (0–14 IU/mL)
Antinuclear antibody IgG	Negative
Anti-mitochondrial antibody	Negative
Anti-smooth muscle antibody	Negative
Anti-liver/kidney microsomal antibody	Negative
Anti-parietal cell antibody	Negative
Anti-proteinase 3 antibody	101 IU/mL (<2.0 IU/mL)
Anti-myeloperoxidase antibody	<0.20 IU/mL (<3.5 IU/mL)
Anti-glomerular basement membrane antibody	Negative
Antineutrophil cytoplasmic antibiotic (ANCA) screen (indirect immunofluorescence)	Positive C-ANCA
Anti-cyclic citrullinated peptide	0.5 U/mL (<5 U/mL)
QuantiFERON	Indeterminant
Procalcitonin	0.18 ng/mL (0.0–0.06 ng/mL)
Sputum culture	Growth of commensals
Q fever serology	Negative
Vitamin D	63
Total protein	69 g/L (60–80 g/L)
Albumin	38 g/L (35–50 g/L)
Protein electrophoresis	Alpha-globulins increased (acute-phase response)
Immunofixation	No paraprotein seen
IgG	12.97 g/L (7.00–16.00 g/L)
IgM	0.73 g/L (0.40–2.30 g/L)
IgA	2.03 g/L (0.70–4.00 g/L)
Leptospira IgM	Negative
Mycoplasma pneumoniae IgM	Negative
Mycoplasma pneumoniae IgG	Negative
Albumin/Creatinine ratio	2.4 mg/mmol (0.0–3.5 mg/mmol)
Vastus lateralis right thigh biopsy	No evidence of myositis or vasculitis
Bronchial washing culture	Acid-fast negative/tuberculosis culture negative/no growth
Bronchoscopy	Normal airways

**Figure 4:**
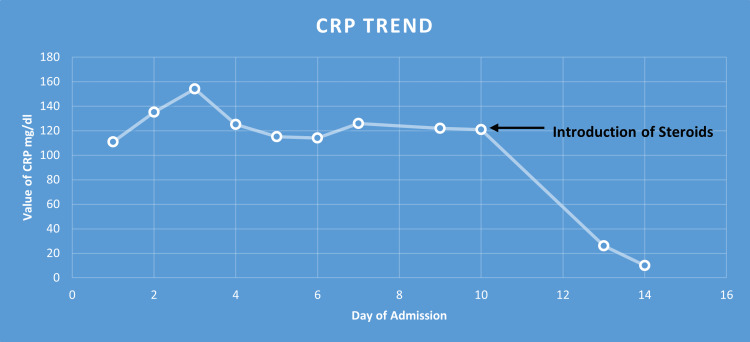
Trend of C-reactive protein with the introduction of steroids.

The patient showed a very good response to steroids, and her CRP markers and temperature spike resolved (Figure 4).

Follow-up

After two weeks of inpatient stay, the patient was discharged on a tapering dose of steroids and followed up in the medical clinic two weeks after discharge. The patient’s repeat blood tests showed normal CRP. Her symptoms of malaise, lethargy, and temperature improved markedly. A week later, the patient was followed up in the rheumatology clinic in the university hospital. Her repeat QuantiFERON was indeterminate again. Her steroid dose was tapered, and she was scheduled for rituximab infusion. Furthermore, she was started on antituberculous medication for prophylaxis against latent tuberculosis.

## Discussion

One of the varied presenting symptoms of GPA can be FUO, as described in case reports in the literature [11-14]. FUO has always been enigmatic for clinicians, as it can be accounted for by more than 200 causal pathologies [15]. In the research conducted within the Infectious Diseases-International Research Initiative (ID-IRI) international clinical research platform on FUO patients with erthyrocyte sedimentation rate >100 mm/hour, infections were responsible for 53% of the cases, neoplasms for 10%, and non-infectious inflammatory diseases (NIIDs) for 9.3% [16]. In another study including 853 FUO patients, 17.94% were caused by NIIDs, which included 1.17% vasculitis cases, and none of them was GPA [17]. Furthermore, a pooled analysis of more than 850 FUO cases showed GPA incidence to be only 0.3%, highlighting that FUO is a rare presenting symptom [18].

GPA (formerly Wegner’s disease) [19] is a small-vessel vasculitis in the ANCA-associated vasculitis category. The prevalence of GPA is 2.3 to 146 cases per million per person. [20] In the majority of reports, the most common age of occurrence of disease is around 45 years. [21] The initial presenting complaint may be non-specific malaise, fever, or weight loss [22]. When the disease is diagnosed, the majority of patients would have hyposmia, anosmia, and nasal obstruction. Furthermore, 50-90% of patients might have pulmonary manifestations, and some of them may suffer alveolar hemorrhage and respiratory failure [23]. Renal manifestations have a close correlation with disease prognosis [24]. GPA can lead to crescent formation and rapidly progressive glomerulonephritis [25]. If untreated, GPA can be life-threatening. Untreated cases can have an average life span of five months, and fewer than 30% survive up to one year [26]. If properly treated, more than 90% of cases can go into remission, especially in the absence of renal involvement [27].

In our case, the patient presented with FUO with non-specific symptoms and had a cavitating lesion in the lung, but no active pulmonary hemorrhage, and there was no involvement of the kidneys. She also complained of nasal stuffiness, but there was no active lesion in the brain and sinus CT. Moreover, examination of the nasal cavities did not reveal any epistaxis or crusting lesions. According to the treatment algorithm recommended by the British Society of Rheumatology, a patient with a life- or organ-threatening disease should be treated for induction of remission with a combination of glucocorticoid (or avacopan) and rituximab/cyclophosphamide [28]. After induction of remission with cyclophosphamide- or rituximab-based therapy, the maintenance of remission is preferably recommended with rituximab-based therapy [27]. In our case, the patient was initially treated with IV glucocorticoids as an inpatient and was later referred to the rheumatology clinic in Model 4 Hospital, where she was started on rituximab infusion, and glucocorticoids were tapered off.

## Conclusions

GPA is an ANCA-associated small-vessel vasculitis affecting multiple organs. It can present with symptoms of FUO. Given the serious implications of delayed diagnosis and missed untreated cases, our case highlights that it is important to consider vasculitis, especially GPA, as an early differential during the workup of FUO.
